# Displacement of Centre of Pressure during Rehabilitation Exercise in Adolescent Idiopathic Scoliosis Patients

**DOI:** 10.3390/jcm10132837

**Published:** 2021-06-27

**Authors:** Luca Marin, Adam Kawczyński, Vittoria Carnevale Pellino, Massimiliano Febbi, Dario Silvestri, Luisella Pedrotti, Nicola Lovecchio, Matteo Vandoni

**Affiliations:** 1 Laboratory for Rehabilitation Medicine and Sport (LARMS), 00133 Rome, Italy; luca.marin@unipv.it (L.M.); massimilianofebbi@gmail.com (M.F.); 2 Department of Research, ASOMI College of Sciences, 2080 Marsa, Malta; direttore@asomi-osteopatia.com; 3 Department of Paralympics Sports, University School of Physical Education, 51-617 Wroclaw, Poland; kawczynski.a@gmail.com; 4 Laboratory of Adapted Motor Activity (LAMA), Department of Public Health, Experimental and Forensic Medicine, University of Pavia, 27100 Pavia, Italy; vittoria.carnevalepellino@unipv.it (V.C.P.); matteo.vandoni@unipv.it (M.V.); 5 Department of Industrial Engineering, University of Tor Vergata, 00133 Rome, Italy; 6 Department of Pediatric Diagnostic Surgical Clinical Science, Section of Pathologies of the Musculoskeletal System, Orthopedics Unit, University of Pavia, 27100 Pavia, Italy; luisella.pedrotti@unipv.it

**Keywords:** adolescent idiopathic scoliosis, posture, balance, center of pressure, self-elongation

## Abstract

Background. Adolescent idiopathic scoliosis (AIS) is included into the category of pathologies that could affect postural control. Rarely AIS shows symptoms but often compromises the normal positioning of the head, trunk and, more generally, of the limbs in the space. We used a stabilometric platform to evaluate the motor control outcomes during a self-elongation in girls with AIS. Methods. In 10 girls with AIS, we evaluated the center of pressure (COP) modifications on a baropodometric platform in a standing position and after a self-elongation correction. Results. All the outcomes (except the eccentricity) showed an increasement during the self-elongation exercise even if the significant differences were not found. Conclusion. SE correction contributes to ameliorate the COP symmetry with a better repercussion on the balance management. This highlights the importance of repetitions during all activities of daily life.

## 1. Introduction

Scoliosis is a three-dimensional spine deviation that is clinically recognized when the deviation on the coronal plane exceeds 10° Cobb. Scoliosis is 20% secondary to another pathology such as cerebral palsy or muscular dystrophy and injuries or infections of the spine, and 80% idiopathic, that means of unknown etiology [1]. Adolescent idiopathic scoliosis (AIS) develops during the adolescent period generally with a deformation peak between 11 and 14 years and a higher prevalence in females (ratio of 1.4:1 for curves between 10° and 20° Cobb and 7.2:1 for curves greater than 40°) [1,2].

Rarely is it symptomatic [1] but in severe cases it can lead to back pain, breathing difficulties due to the deformation of ribs, and morphological changes of the torso that affect physical appearance. Subjects affected by AIS usually show also psychological and social difficulties such as depression and lack of self-esteem [3,4,5]. Moreover, AIS is included into the category of pathologies that could affect postural control and balance as well and indirectly modifies the center of mass (COM) displacement [6]. In fact, the common spatial position of the body segment, especially the head and trunk, can be modified by scoliosis [7], or by the involuntary arrangement of the subjects [8] who alter posture to counteract scoliosis [9], because of the spontaneous correction of the control system to keep the eyes aligned with the horizontal line. For these reasons, spinal alignment falls further altering scoliotic curves. Thus, AIS could be considered a pathology that alters subjects’ postural control [10], leading to a reduction in the capacity of the postural control [11] as well as during the motor response to an external stimulus [12].

The most used method to evaluate the postural control is the assessment of center of pressure (COP) [13,14,15,16,17], that is, the point at which the ground reaction forces are considered to act [10,11,18]. In point of this, the COP displacement is used to estimate the sway of the COM during physical activities such as everyday life gestures or sport-specific performance [11,19]. COP sway, actually, is considered to assess static posture [20], the effects of different dental occlusion conditions [13,21,22], the outcomes after session of physical therapy [6] or in consequence of fatiguing [7]. In particular, in AIS subjects, COP sway assessment could ameliorate the general evaluation of the motor control of the subjects and then improve the personalized exercise routine. 

The use of a stabilometric platforms (the most reliable instruments used for postural assessment) [10,23,24] could be also used to evaluate the motor control (outcomes) of AIS young during specific active exercise performed to reduce the curve (i.e., self-elongation).

Indeed, self-elongation (SE), as suggested by many rehabilitation methods, can be defined as a self-correction exercise with an active recruitment of the trunk muscles aimed to temporarily re-arrange the vertebrae in a straight position. Often, self-correction is recommended to patients as a routine exercise during daily life activities [10,25]. In particular, once the patient is aware of his curves displacement and the required changes to correct it, the patient is able to consciously find the best possible alignment of the spine. The SE main goal is to help muscle activation improving spinal stability with the effect of developing postural control, vital capacity and quality of life [25]. 

Thus, the aim of this study was the evaluation of the changes of COP during specific SE exercise to verify if the correction of the alignment of the spine had a secondary benefit correlated to body balance arrangement.

## 2. Materials and Methods

### 2.1. Subject

A convenient sample of 10 girls (age 13–15 years) with a diagnosis of AIS were freely recruited during the weekly routine rehabilitation session. All patients had a diagnosis of primary thorax scoliosis defined as right convex and a frequency of clinic gym visitation of twice weekly. They were under physiotherapist control from 28 months (on average).

Anthropometric measurements were performed as described elsewhere [26]. Weight was measured with participants not wearing shoes and in light clothing, standing upright in the center of the scale platform (Seca, Hamburg, Germany) facing the recorder, hands at sides, and looking straight ahead. Standing height was measured using a Harpenden stadiometer (Holtain Ltd., Cross-Well, UK) with a fixed vertical backboard and an adjustable head piece. The measurement was taken on the subjects in an upright position, without shoes, with their heels together and toes apart, hands at sides, aligning the head in the Frankfort horizontal plane. Two measurements were taken for each parameter, and a third was obtained if a discrepancy was noted between the initial measurements for weight (>500 g) and height (>0.5 cm). Final growth parameter values were based on the average of the two closest measurements. Cobb angle [27] is measured on a posterior-anterior or anterior-posterior x-ray by drawing a line at the top of the vertebra (above a curve’s apex) with the greatest lateral tilt and another line at the bottom of the vertebra (beneath the same curve’s apex) with the greatest lateral tilt. Extend the lines to the margin of the image, then draw, respectively, the third and fourth perpendicular lines. The Cobb angle is calculated where the lines intersect. 

The Risser sign [28] is determined from an x-ray image of the pelvis and references the appearance of a crescent-shaped line of bone formation—visualize a mushroom cap—which appears across the top of each side of the pelvis on an anterior-posterior or posterior-anterior x-ray image. This measurement is used to evaluate the skeletal maturity or completed growth, graded on a scale from 0–5 (0 indicates much potential growth remains and 5 indicates skeletal maturity). All data are shown in Table 1. 

Parental consents were obtained before participation in the study, completing a secondary anamnesis to verify other impairments or psychological transitory stress that could affect the compliance of the subjects.

All procedures were in accordance with the Declaration of Helsinki (1975) as revised in 2008 and were preventively approved by the clinic ethic committee (Area Vasta Pavia’ Ethical Committee Protocol code 20180036031).

### 2.2. Instruments 

To evaluate patients’ COP, a baropodometric platform with resistive 24 k gold-coated sensors was used (FreeMed^®^, Sensormedica, Roma, Italy). Dimensions were 120 × 50 cm and the use of a sampling frequency up to 400 Hz guaranteed a high level of accuracy during the stance phase (Intra Class Correlation between 0.80 and 0.83; [24]). Acquired data were processed by a computer using a specific software with an integrated module that allows different acquisitions into a single database. Moreover, the software in real time (during acquisitions) showed the total load pressures and the single contribution by the two feet.

### 2.3. Procedures

Body sway was assessed in the afternoon (4–5:00 or 5–6:00 p.m.) according to the scholastic obligations of the students; in the same clinical setting with a constant temperature of 21 centigrade. All AIS subjects stood barefooted over the stabilometric platform: Subjects were asked to keep their feet in contact with the platform, hang their arms along the trunk; look straight ahead toward a mirror 3 m far to avoid movement response [17]. In particular, AIS subjects kept the standing position in natural way for 10 s (without modification of the feet position and the general arrangement; [29] while the second recording phase was immediately recorded with the subject performing SE according to the procedure learned with the physical therapist. In this case, the monitor was covered to guarantee a freely execution avoiding visual influence by the feedback of the feet pressure.

SE exercise is performed with the auto-correction of the trunk-alignment and spinal deforment with a specific focus on postural balance and proprioception. In particular, the general routine of exercise was composed by four to six exercises: upwards spine elongation; major curve translation on the frontal plane, towards the side of the concavity; vertebral rotation on the horizontal plane; thoracic kyphotization (when necessary); lordosization of the lumbar spine (when necessary). All the subjects performed four exercises maintaining the correct position for at least 15 s with a short rest time (up to 7 s) between exercises. The last two exercises were performed only if deemed necessary from the physiotherapist. 

All the SE sessions were conducted by the same examiner in the same laboratory environment to minimize any noise or other disturbances. 

### 2.4. Statistical and Data Analysis

The software provided the maximum display of the COP (mm) along the X (medio-lateral direction) and Y (antero-posterior direction) axis (sway-X and Sway-Y) such as the total area (sway area, mm^2^) and the speed of the COP displacement (sway speed; mm/s). In particular, the eccentricity value of the ellipse (*e*) was extracted as an index of the symmetry of the displacement of the COP. This value defines the shape skewness of an ellipse considering the length of the two axes. This value ranges between 0 and 1; whereas if the index corresponds to zero the ellipse is a circle while in case of *e* = 1 the ellipse corresponds to a line.

All the quantitative variables are shown as median and IQR. All the qualitative variables are shown as percentage when appropriate. We tested the normality using Shapiro-Wilk test. To evaluate the differences between the two conditions (standing, SP; and during self-elongation, SE) data were matched and compared with a non-parametric approach through Wilcoxon signed-rank test. The significance was set at a p-value less than 0.05 and eventually the Effect Size (ES) used to verify the biological consistence of the differences. Statistical analyses were performed using The Jamovi project (2021): *jamovi* Version 1.6 for Mac, Sydney, Australia [Computer Software]. 

## 3. Results

COP sway outcomes are described in Table 2. In general, all the outcomes (except the eccentricity, e) showed an increasement during the self-elongation exercise, even if the significant differences were not found. The variation along X and Y axis were very similar (20%) resulting in important Sway Area (variation of 50%). During SE, the Speed Sway increased per 1 mm/s that correspond at 14%. The e index decreased showing a moderate value of ES = 0.35. 

## 4. Discussion

In the last years, the improvement of technologies such as baropodometric platforms helped physiotherapists to ameliorate the evaluation and then the treatment of pathologies that could affect postural control and balance. In particular, the aim of this study was to assess COP sway in adolescents with AIS during SE correction, because AIS subjects’ postural sway is lower than healthy subjects and become wider when performing dynamic adaptations [12,30,31].

Our results showed a small increment in Sway-X, Sway-Y, Sway Area and Speed Sway that could be interpreted as a negative outcome from an early evaluation. Instead, it is necessary to consider all the modalities of movement as a confounder for COP arrangement. For example, Pasha et al. [31] found, in a sample of AIS subjects, significant differences in the distribution of the COP due to upper limbs modified position. Even if the SE appears as an undetectable movement, it involved a deep variation [32] of the spine that depends on the linear combination of the pelvic incidence, maximum lumbar lordosis, sacral slope, the pelvic tilt and the thoracic kyphosis [33]. Moreover, if experienced therapists would consider that, in AIS subjects, the frontal Cobbs, apical rotations, kyphosis, pelvic tilt, and sacral slope could affect the postural balance [31], then the increment of the sway indexes are not unexpected. In fact, previously, [34] stressed the importance of the rearrangement of the motor system (i.e., COP position) with a different representation of the body in the space for AIS subjects that, if negatively integrated, could lead to maladjustments [35,36].

Considering these assumptions, the sway trend could be considered as a modification of the motor control in patients during SE performance; complex and non-immediate adaptations seem to be promising to enhance postural control [12]. In this case, we observed an initial re-arrangement of body in the space (sway indexes increased) with partial decrease of *e* index (possible sign of better balancing), whereas the oscillation in medio-lateral and antero-posterior direction become similar thanks to a more rounded Sway Area. Our results underlined that the postural control in adolescents with AIS tends to modify through SE exercises and the subsequent body arrangement. In fact, proprioceptive and visual information changed with a shift to a new asset. These changes in torso asset and in head-neck position affected COP sway, which tends to equalize the two axes.

We are conscious that this study had some limitations. First, the small sample of recruited subjects did not allow us to perform a more in-depth analysis. Nevertheless, participants were all young girls with similar deviation contributing to homogenize the data and to exclude bias caused by gender differences. Finally, the novelty of this work does not only consist in the evaluation of the scoliotic curve correction level but mainly in measuring the variations of the COP (i.e., balance) displacement during a physical correction.

## 5. Conclusions

In conclusion, we stated that SE correction could contribute to ameliorate the COP symmetry with a possible better repercussion on balance management. This highlights the importance of the implementation of postural control obtained by SE correction during all activities of daily life.

## Figures and Tables

**Table 1:** Anthropometric characteristic of the convenient sample of girls.

Subject	Age (Years)	Weight (kg)	Height (cm)	BMI	Risser	Cobb (Degrees)
F1	14	52	158	20.83	3	12
F2	15	57	165	20.94	3	12
F3	14	55	160	21.48	3	13
F4	14	48	158	19.23	5	18
F5	13	47	145	22.35	4	15
F6	13	47	151	20.61	3	12
F7	13	42	149	18.92	3	13
F8	14	50	155	20.81	4	15
F9	13	53	159	20.96	3	13
F10	15	58	166	21.05	5	18
mean	13.8	50.9	156.6	20.7	3.6	14.1
SD	0.8	5.0	6.7	1.0	0.8	2.2

**Table 2:** The sway parameter of the Centre of Pressure (COP) in standing position (SP) and during the performance of self-elongation (SE). All values are shown as median and IQR.

	Sway-X (mm)		Sway-Y (mm)		Sway Area (mm^2^)		Speed Sway (mm/s)		*e*	
	SP	SE	SP	SE	SP	SE	SP	SE	SP	SE
Median value	3.91	4.73	4.89	5.82	20.18	32.09	8.65	9.87	0.50	0.36
*p*-value	0.99		0.68		0.69		0.62		0.37	
Effect size	−0.01		−0.16		−0.16		−0.20		0.34	
Q1	3.38	3.06	3.85	4.73	11.53	13.55	7.13	8.17	0.30	0.16
Q3	5.91	7.51	8.13	8.2	34.77	46.76	11.62	11.62	0.74	0.55

Legend: Q1 = 25th quartile; Q3 = 75th quartile; IQR: interquartile range.

## Data Availability

The data presented in this study are available on request from the corresponding author. The data are not publicly available due to privacy reasons.
